# Treadmill training in Parkinson’s disease is underpinned by the interregional connectivity in cortical-subcortical network

**DOI:** 10.1038/s41531-022-00427-3

**Published:** 2022-11-11

**Authors:** Hao Ding, Amgad Droby, Abdul Rauf Anwar, Manuel Bange, Jeffrey M. Hausdorff, Bahman Nasseroleslami, Anat Mirelman, Inbal Maidan, Sergiu Groppa, Muthuraman Muthuraman

**Affiliations:** 1 Department of Neurology, Focus Program Translational Neuroscience (FTN), University Medical Center of the Johannes Gutenberg University Mainz, Mainz, Germany; 2 Academic Unit of Neurology, Trinity College Dublin, The University of Dublin, Dublin, Ireland; 3 Department of Neurology, Sackler Faculty of Medicine, Tel Aviv University, Tel Aviv, Israel; 4 Sagol School of Neuroscience, Tel Aviv University, Tel Aviv, Israel; 5 Laboratory for Early Markers of Neurodegeneration (LEMON), Center for the Study of Movement, Cognition, and Mobility (CMCM), Neurological Institute, Tel Aviv Sourasky Medical Center, Tel Aviv, Israel; 6 Biomedical Engineering Centre, UET Lahore (KSK Campus), Lahore, Pakistan; 7 Department of Physical Therapy, Sackler Faculty of Medicine, Tel Aviv University, Tel Aviv, Israel; 8 Rush Alzheimer’s Disease Center and Department of Orthopaedic Surgery, Rush University Medical Center, Chicago, IL USA

**Keywords:** Parkinson's disease, Learning algorithms, Cerebellum

## Abstract

Treadmill training (TT) has been extensively used as an intervention to improve gait and mobility in patients with Parkinson’s disease (PD). Regional and global effects on brain activity could be induced through TT. Training effects can lead to a beneficial shift of interregional connectivity towards a physiological range. The current work investigates the effects of TT on brain activity and connectivity during walking and at rest by using both functional near-infrared spectroscopy and functional magnetic resonance imaging. Nineteen PD patients (74.0 ± 6.59 years, 13 males, disease duration 10.45 ± 6.83 years) before and after 6 weeks of TT, along with 19 age-matched healthy controls were assessed. Interregional effective connectivity (EC) between cortical and subcortical regions were assessed and its interrelation to prefrontal cortex (PFC) activity. Support vector regression (SVR) on the resting-state ECs was used to predict prefrontal connectivity. In response to TT, EC analysis indicated modifications in the patients with PD towards the level of healthy controls during walking and at rest. SVR revealed cerebellum related connectivity patterns that were associated with the training effect on PFC. These findings suggest that the potential therapeutic effect of training on brain activity may be facilitated via changes in compensatory modulation of the cerebellar interregional connectivity.

## Introduction

Parkinson’s disease (PD) is a common neurodegenerative disorder that is characterized by a combination of motor and non-motor symptoms^1^. PD manifests clinically with variable degrees of bradykinesia, resting tremor, and muscle rigidity, in addition to posture instability, and gait abnormalities^2^. Gait impairments are common, and gait generally deteriorates over time. As the disease progresses, reduced gait speed, step length and impaired rhythmicity are accompanied by increased instability which severely affect the patient’s independence and quality of life^3^.

Clinical assessments of disease severity such as those based on the Movement Disorder Society-Unified Parkinson’s Disease Rating Scale (MDS-UPDRS) or Hoehn and Yahr (H&Y) staging correlate with the altered brain connectivity between different brain areas^1,4^. Resting-state functional magnetic resonance imaging (rs-fMRI), which measures the blood oxygen level-dependent (BOLD) response, has been increasingly used to quantify the dynamics of neuronal networks among different brain regions. Previous neuroimaging and physiological studies^5,6^ proposed several probable origins of the gait impairments in PD including frontal cortical regions, the basal ganglia, cerebellum and brainstem. However, since rs-fMRI is measured at rest and not during actual gait, it can only provide an indirect reflection of the neural impairments that contribute to gait alterations. Functional near-infrared spectroscopy (fNIRS) applies an optical technique to capture functional hemodynamics^7^ offers a way of assessing cortical activation during actual walking. Studies in patients with PD have shown that under normal regular walking tasks, patients exhibit higher cortical activation of the dorsolateral prefrontal cortex compared with healthy, age-matched older adults^8^. Previous studies further demonstrated that the treadmill training (TT) can improve gait performance^9,10^ and reduce prefrontal activation during a simple walking task^11,12^. In a multi-center study^13^, a TT program for patients with PD showed improvements in both cognitive function and mobility as well as modifications in cerebellum activity^14^. Nevertheless, the patterns of neuronal activity underlying the elevated prefrontal activity remain to be established.

In PD, decreased functional connectivity between subareas in subcortical region and motor cortex were reported^15,16^. By measuring the temporal dependency between the anatomically separated brain areas, the resulting change in brain connectivity can provide insight into the relationship between brain areas^17^.

**Fig. 1:**
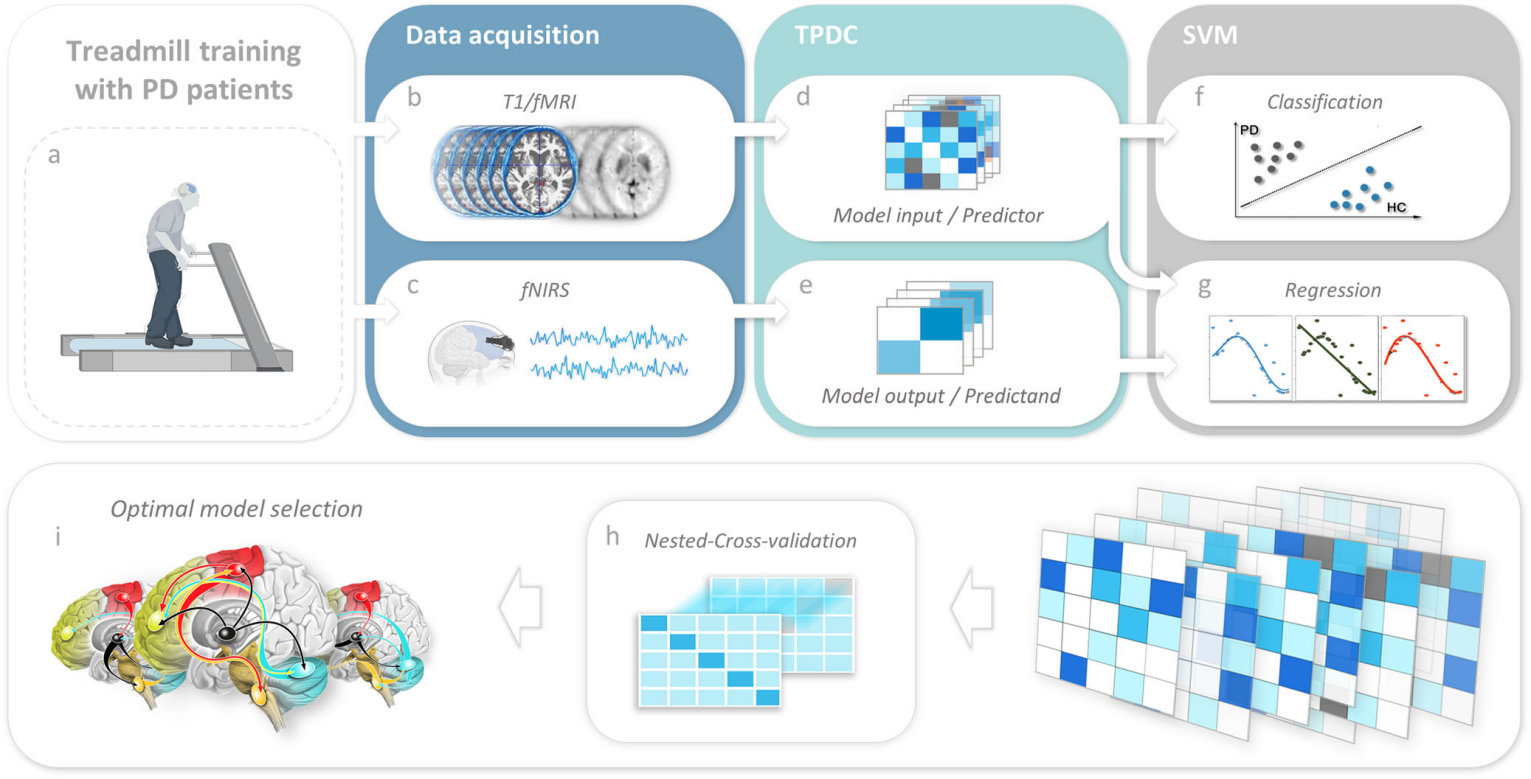
Methodology pipeline. **a** PD patients underwent 6 weeks of treadmill training. **b** T1/fMRI acquisition during resting state before and after the training. **c** fNIRS acquisition during normal and dual-task walking tasks. **d** EC analysis with TPDC for fMRI. **e** EC analysis with TPDC for fNIRS. **f** SVM classification based on rs-fMRI connectivity as the predictors. **g** SVM regression based on rs-fMRI connectivity to predict fNIRS connectivity. **h** All the feature combinations pass through nested cross­ validation. **i** Select the optimal SVM model based on the model performance. PD Parkinson’s disease, fNIRS functional near infrared spectroscopy, EC effective connectivity, fMRI functional magnetic resonance imaging, SVM support vector machine, TPDC time resolved partial directed coherence.

Leveraging the unique perspectives and advantages of rs-fMRI and fNIRS, we conducted an integrated analyses using both imaging techniques to better understand the impact of TT on brain activity in PD. Based on previous research, we focused on large-scale regions of interest (i.e., prefrontal cortex; motor cortex; basal ganglia, brainstem and cerebellum) to gain a conceptual and robust overview of the underlying patterns of altered brain connectivity in PD. We leveraged the directionality of effective connectivity (EC) analysis to reveal important information on the influence from one brain region over another, and adopted support vector regression (SVR) to model the prefrontal activity during walking based on resting-state ECs. By combining different hemodynamic modalities (fNIRS; rs-fMRI), the EC predictors underlying prefrontal activity following TT can be highlighted and provide a better understanding on the cortical-subcortical brain network interactions related to the training effects in patients with PD (Fig. 1).

We hypothesized that EC estimation based on large-scale brain regions will be a sensitive measure of training effects, revealing network patterns of intrinsic connectivity associated with prefrontal activity during walking.

## Results

### Patient characteristics

As shown in Table 1, no differences in age were observed between groups. Patients with PD had lower scores on Montreal Cognitive Assessment (MoCA) and longer completion time on Trial Making Tests (TMT), compared with the healthy older adults. As expected, patients with PD showed shorter gait speed and stride length.

**Table 1:** Demographics and disease characteristics at baseline.

Characteristic	PD *n* = 19	HC *n* = 19	*P*-value
Mean age, years	74.0 (6.59)	69.86 (5.87)	0.06
Sex, *n* (%)			0.33
Male	13 (68)	10 (53)	
Female	6 (32)	9 (47)	
Disease duration, years	10.45 (6.83)	N/A	
MOCA	22.15 (3.28)	27.37(2.19)	<0.001
UPDRS-III score	31.65 (14.61)	N/A	
UPDRS-Total score	71.50 (26.26)	N/A	
TMT­A, second	106 (51.68)	60.79 (22.31)	<0.001
TMT­B, second	193.22 (82.5)	113.53 (27.23)	<0.001
NW gait speed, cm/s	92.39 (34.45)	106.91 (12.02)	0.11
DT gait speed, cm/s	87.69 (27.74)	98.61 (18.99)	0.19
NW stride length, m	1.14 (0.12)	1.22 (0.10)	0.10
DT stride length, m	1.17 (0.11)	1.18 (0.14)	0.88

*PD* Parkinson’s disease patients, *HC* healthy controls, *TMT* trail making test, *NW* normal walking, *DT* dual-task walking, *UPDRS* unified Parkinson’s disease rating scale, *MOCA* the Montreal cognitive assessment, (#) standard deviation.

### The effects of treadmill training on clinical assessments and gait performance

After TT, better cognitive and gait performance were observed. The cognitive assessments such as TMTa and TMTb showed reduced completion time (*p* < 0.001 and *p* = 0.002 respectively), and gait speed increased during both normal walking (NW) (*p* = 0.008) and dual-task walking (DT) (*p* = 0.005). Longer stride length and stride time along with reduced cadence were observed after training in the DT walk (Table 2).

**Table 2:** Gait and cognitive performance scores of the PD group before and after TT.

Characteristic	PD baseline(t1)	PD follow-up(t2)	Δ(t2-t1)	*P*-value
TMT­A, second	106 (51.68)	107.77 (62.44)	1.77 (39.56)	<0.001*
TMT­B, second	193.22 (82.5)	180.4 (107.7)	−12.82 (73.7)	0.002*
NW gait speed, cm/s	92.39 (34.45)	95.25 (32.97)	2.86 (29.2)	0.008*
NW stride length, m	1.14 (0.12)	1.11 (0.12)	−0.03 (0.15)	0.031†
NW stride time, second	4.8 (1.88)	4.56 (2.29)	−0.24 (2.7)	0.73*
NW cadence, steps/min	104.17 (11.27)	107.63 (14.32)	3.46 (13)	0.26*
DT gait speed, cm/s	87.69 (27.74)	90.83 (28.02)	3.14 (22.2)	0.005*†
DT stride length, m	1.17 (0.11)	1.19 (0.13)	0.02 (0.1)	0.021*†
DT stride time, second	5.6 (1.9)	6.38 (5.16)	0.78 (4.37)	0.063*
DT cadence, steps/min	100.82 (11.5)	98.93 (11.46)	−1.89 (10.6)	0.063*

*PD* Parkinson’s disease patients, *TMT* Trail Making Test, *NW* normal walking, *DT* dual task walking.

(#) standard deviation; * Paired-samples *t*-test, † not significant after Bonferroni correction for multiple comparisons.

### Reduced prefrontal activity during walking after treadmill training

**Fig. 2:**
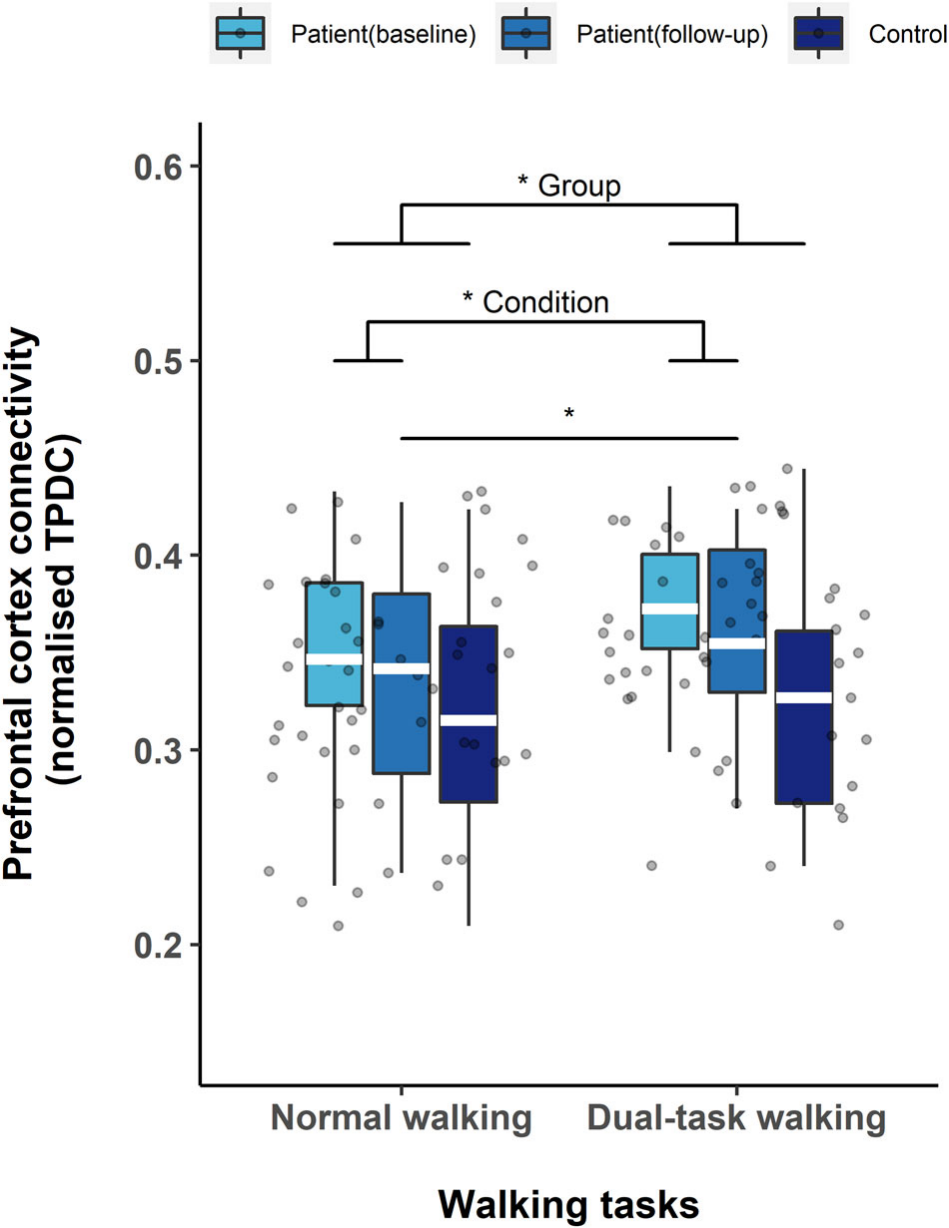
Effective connectivity in prefrontal cortex during walking tasks. Effective connectivity value in prefrontal cortex (vertical axis) during normal walking and dual-task walking conditions (horizontal axis). The box represents lower quartile, median and upper quartile. Non-parametric two-way ANOVA indicated significant main effect for training (between patients and controls at baseline) and condition (between NW and DT). Wilcoxon signed-rank test indicated significant difference between NW and DT walking in patients at follow-up. ^∗^*p* < 0.05; white line (within the box): median level.

Figure 2 illustrates the prefrontal connectivity for patients at baseline, follow-up and for controls during both walking tasks. A non-parametric two-way ANOVA revealed a significant main effect (*F* = 6.955; *p* = 0.012) between patients and controls at baseline, whereas no significance was found for the condition factor (*F* = 1.500; *p* = 0.228) between NW and DT, or interaction effect (*F* = 0.720; *p* = 0.401). Regarding the training effect in the patients, non-parametric ANOVA revealed a significant condition difference (*F* = 6.476; *p* = 0.013) between patients at baseline and follow-up, whereas there was no significance found for training factor (*F* = 1.750; *p* = 0.191) and interaction effect (*F* = 0.021; *p* = 0.884). Further, post-hoc Wilcoxon signed-ranks test showed no significant difference between NW and DT walking (*p* = 0.123) in the patient group at baseline. At follow-up, the patients showed a significant lower prefrontal connectivity during NW compared with DT (*p* = 0.018).

### Training effects on resting-state brain activity

**Fig. 3:**
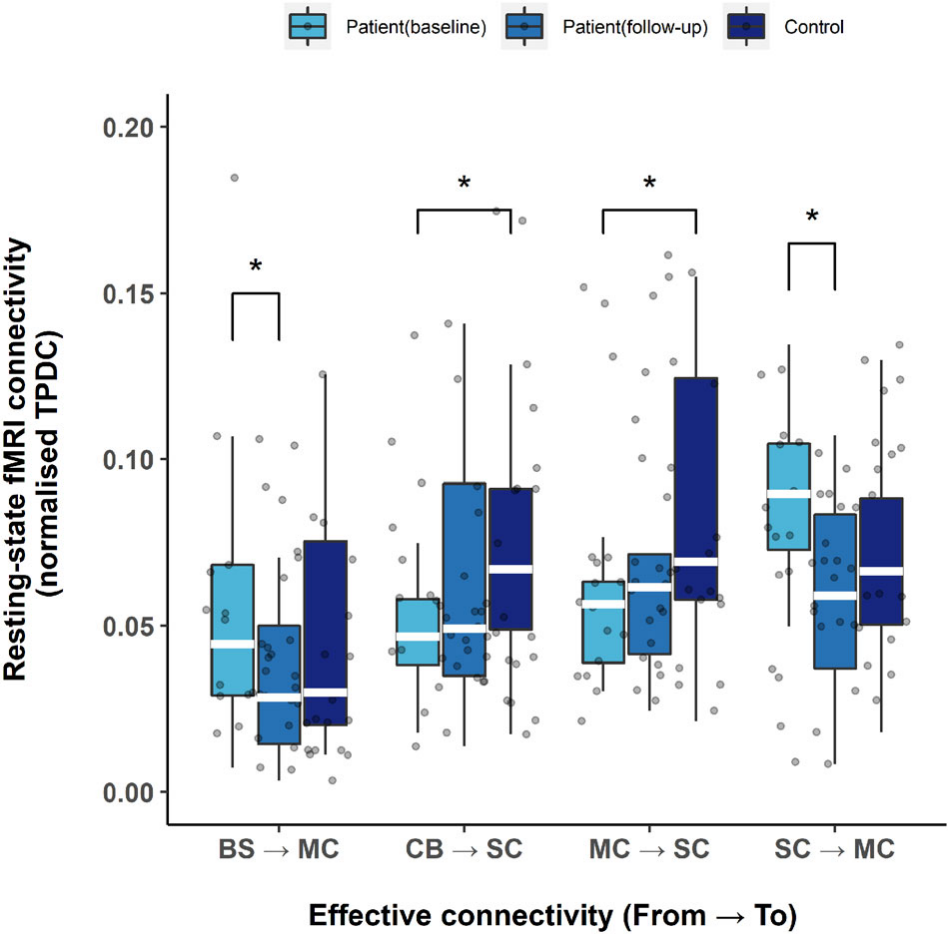
Training effects on resting-state effective connectivity in Parkinson’s disease patients. Effective connectivity value during resting-state between the patients before and after treadmill training. The box represents lower quartile, median and upper quartile. The control group served as reference to the effects of training. BS brainstem, CB cerebellum, MC motor cortex, SC subcortical region, TPDC time-resolved partial directed coherence. *p < 0.05; white line (within the box): median level.

To determine whether resting-state ECs differ in patients before and after TT as well as in comparison with healthy controls, Mann–Whitney U test was performed on each EC between patients at baseline and healthy controls, showing a significantly lower level of connectivity: *cerebellum* ⇀ *subcortical region* (*p* = 0.023) and *motor cortex* ⇀ *subcortical region* (*p* = 0.049). Following TT, these levels of connectivity increased in patients towards the levels found in healthy controls, and no significant differences were found between patients at follow-up and healthy controls (*p* > 0.05, see Supplementary Table S2). Based on Wilcoxon signed-ranks tests, patients at follow-up demonstrated a significantly reduced level of connectivity compared with baseline *brainstem* ⇀ *motor cortex* (*p* = 0.044) and *subcortical region* ⇀ *motor cortex* (*p* = 0.044) as shown in Fig. 3. There was no statistical significance found in other ECs. The detailed results are summarized in the supplementary material (Supplementary Table S2).

For further assessment of the relationship in the above-mentioned significant observations among patients, Spearman’s correlation was performed on the difference of ECs in patients before and after TT by subtracting the EC values between baseline and follow-up, as noted by ΔECs.

**Fig. 4:**
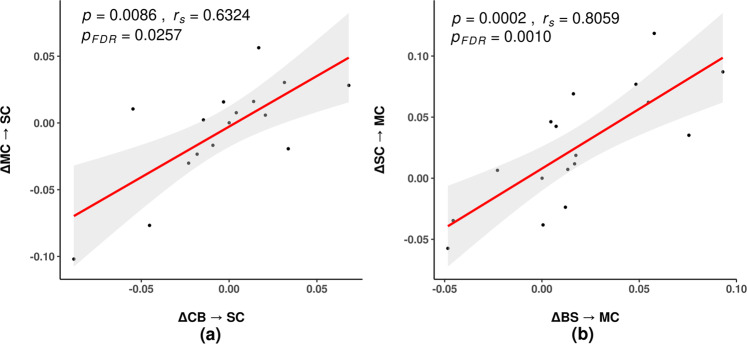
Correlation between ∆EC in PD patients. ∆EC indicates the difference of the EC connectivity before and after TT. **a** ∆EC correlation between *CB*⇀*SC* and *MC*⇀*SC*. **b** ∆EC correlation between *BS*⇀*MC* and *SC*⇀*MC*. BS brainstem, CB cerebellum, MC motor cortex, SC subcortical region, TT treadmill training, EC effective connectivity, ∆ difference of EC in the patients at baseline and follow-up, FDR false discovery rate.

Figure 4 shows correlation analysis between ΔECs with false discovery rate (FDR) correction. Both subcortical related ECs (*cerebellum* ⇀ *subcortical region*; *motor cortex* ⇀ *subcortical region*), which exhibited a lower level of connectivity compared with controls (Fig. 3), showed strong and positive correlations. (*r*_s_ = 0.6324, *p* = 0.0086, *p*_FDR_ = 0.0257). The other two motor related ECs (*brainstem* ⇀ *motor cortex; subcortical region* ⇀ *motor cortex*), which showed higher level of connectivity compared with controls (Fig. 3), were also found to be positively correlated (*r*_s_ = 0.8059, *p* = 0.0002, *p*_FDR_ = 0.0010).

### Association between intrinsic brain connectivity and motor performance

**Fig. 5:**
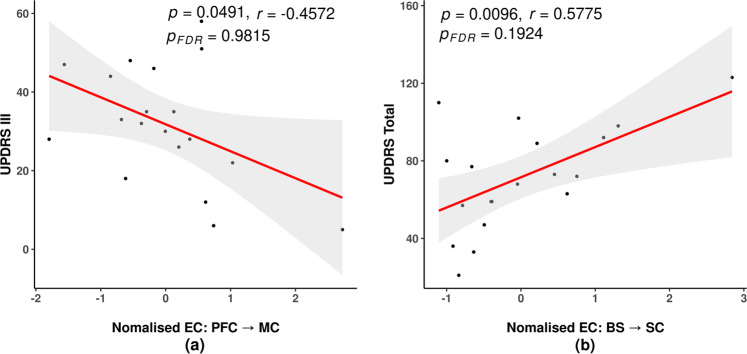
Correlation between resting-state EC and UPDRS scores in PD patients. **a** EC: *PFC*⇀*MC* in relation to UPDRS-III. **b** Correlation between EC: *BS*⇀*SC* and UPDRS-Total scores. BS brainstem, CB cerebellum, MC motor cortex, PFC prefrontal cortex, SC subcortical region, EC effective connectivity, UPDRS unified Parkinson’s disease rating scores, FDR false discovery rate.

We examined the correlation between brain connectivity during resting state and clinical parameters including UPDRS-III and UPDRS-Total scores in the patient group at baseline. Figure 5a shows a moderate negative correlation between UPDRS-III and connectivity in *prefrontal cortex* ⇀ *motor cortex* (*r* = −0.4572, *p* = 0.0491, *p*_FDR_ = 0.9815), indicating that a better motor performance is related to higher connectivity from prefrontal cortex to motor cortex. Moreover, a positive correlation was observed between UPDRS-Total scores and connectivity *brainstem* ⇀ *subcortical region* (*r* = 0.5775, *p* = 0.0096, *p*_FDR_ = 0.1924, Fig. 5b). There was no statistical significance found between UPDRS and the rest of ECs. The detailed results are summarized in the supplementary material (Supplementary Table S3). Following the FDR correction, the p-values are not statistically significant.

### Resting-state ECs underlying prefrontal activity during walking

**Fig. 6:**
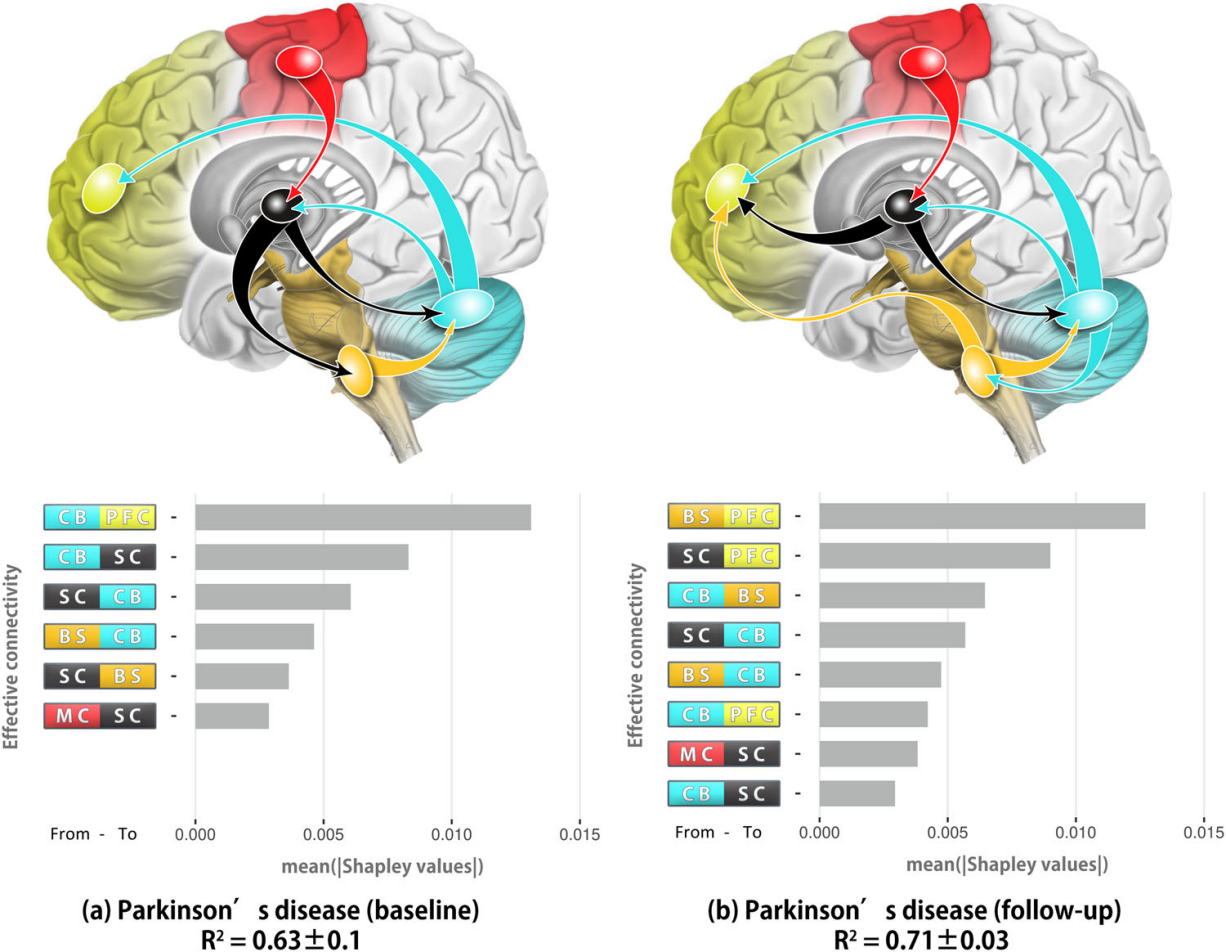
Optimal SVR models for patients before and after treadmill training. **a** Optimal SVR model for patient group at baseline. **b** Optimal SVR model for patient group at follow-up. Feature contributions (gray bars) are listed below the corresponding model. PFC prefrontal cortex, MC motor cortex, BS brainstem, CB cerebellum, SC subcortical region.

Based on the observation that the patients exhibited reduced EC in the prefrontal cortex during NW compared to DT at follow-up (Fig. 2), we further investigated which resting-state ECs were related to the changes in the prefrontal cortex. We assessed this information by comparing optimal SVR models trained on the resting-state ECs before and after TT in the patients. Figure 6 shows the models for both baseline and follow-up that outperformed other feature combinations. The prediction accuracy of *R*-squared are similar (baseline: *R*^2^ = 0.63 ± 0.1; follow-up: *R*^2^ = 0.71 ± 0.03): the optimal model for baseline patients was trained with 6 EC features whereas the model for follow-up was trained with 8 EC features. At follow-up, the patients demonstrated a similar EC pattern compared to the baseline with differences from *subcortical region* ⇀ *brainstem* (Fig. 6a) switched to *subcortical region* ⇀ *prefrontal cortex*, *brainstem* ⇀ *prefrontal cortex* and *cerebellum* ⇀ *brainstem* (Fig. 6b). According to the feature contributions, cerebellum related connectivity was still closely related to prefrontal activity, but *cerebellum* ⇀ *prefrontal cortex*, which contributed greatly at baseline, was less informative in this pattern.

### Effective connectivity patterns that discriminate Parkinson’s disease patients from controls

**Fig. 7:**
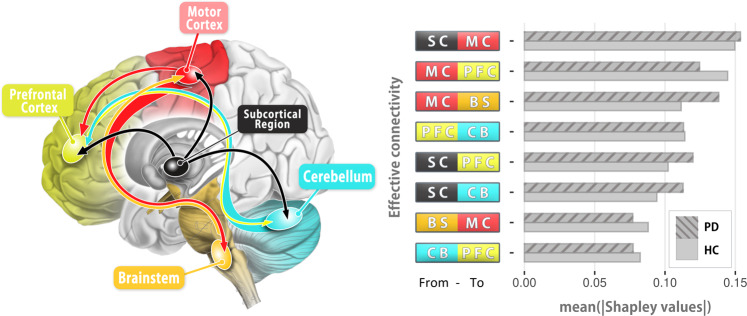
Optimal SVM classifier and corresponding Shapley value as the feature contribution. The optimal SVM classification based on EC features reached 91.05% mean accuracy; mean sensitivity: 93.68% ± 3.9; mean specificity: 88.42% ± 7 in classifying patients at baseline and controls. The EC features and their Shapley value as the contribution to the model prediction are shown in the bar graph (right). Shapley values are ranked in descending order by the average of both groups. PFC prefrontal cortex, MC motor cortex, BS brainstem, CB cerebellum, SC subcortical region, PD Parkinson’s disease patients at baseline, HC healthy controls.

The optimal support-vector machine (SVM) classifiers were able to differentiate Parkinson’s disease patients and controls with mean accuracy ± standard error: 91.05% ± 2; mean sensitivity: 93.68% ± 3.9; mean specificity: 88.42% ± 7. Figure 7 demonstrated the optimal model and the EC features with its contribution (as quantified by the Shapley values) to the model prediction. The classifiers were trained on 8 ECs (*motor cortex* ⇀ *prefrontal cortex*, *motor cortex* ⇀ *brainstem*, *subcortical region* ⇀ *motor cortex*, *subcortical region* ⇀ *prefrontal cortex*, *subcortical region* ⇀ *cerebellum*, *prefrontal cortex* ⇀ *cerebellum*, *brainstem* ⇀ *motor cortex*, *cerebellum* ⇀ *prefrontal cortex*). This EC pattern is distinct because this specific combination outperformed all other feature combinations. A higher number of feature combinations did not yield better accuracy. In terms of feature contribution, the Shapley value was calculated per each observation and summarized into groups. *Subcortical region* ⇀ *motor cortex* shows the highest contribution to the classified outcome for both patient and control groups, being the most informative feature among the predictors. The major contributions within the network can be considered from the second feature (*motor cortex* ⇀ *prefrontal cortex*) to the sixth feature (*subcortical region* ⇀ *cerebellum*) since these features have similar range of scale and are more informative in distinguishing the patient group, but *motor cortex* ⇀ *prefrontal cortex* contributed to the prediction of the control group more than the patient group. *Brainstem* ⇀ *motor cortex* and *cerebellum* ⇀ *prefrontal cortex* contributed the least among all the features and both are more informative in distinguishing the control group.

## Discussion

To our knowledge, this work is a pioneering study utilizing a combined approach hemodynamic fNIRS and fMRI to investigate the EC patterns underlying prefrontal activity during walking in PD. We investigated the EC modifications in patients with PD, compared with age matched healthy controls using an intervention design. We found reduction of EC in prefrontal cortex during walking and unidirectional ECs in *brainstem* ⇀ *motor cortex* and *subcortical region* ⇀ *motor cortex* during resting-state. These modifications showed a trend towards the level of the control group, suggesting patterns of neural changes following TT. Furthermore, the cerebellum and its intrinsic connectivity with the brainstem and subcortical region were the main features of a specific EC pattern associated with the prefrontal cortex during walking in the patients group. After TT, the prefrontal and brainstem directed connectivity (*brainstem* ⇀ *prefrontal cortex* and *subcortical region* ⇀ *prefrontal cortex*; *cerebellum* ⇀ *brainstem* and *subcortical region* ⇀ *brainstem*) present as the newly emerged features in the pattern, which might be reflective of the brains’ compensation to the reduced prefrontal activity following TT. Based on these results, we further tested the sensitivity of EC estimation based on large-scale brain regions via classification method and obtained 91.05% mean accuracy.

In PD patients, the dopaminergic inputs to the striatum are diminished due to neuronal degeneration in substantia nigra pars compacta, making it difficult to generate the transient inhibition to the globus pallidus^18^. It increases the tonic inhibition from the internal segment of the globus pallidus to the thalamus, resulting in thalamic excitation of the motor cortex less likely^18^. This impairment in the cortico-basal ganglia circuits may imply the need for compensation. Recent studies found elevated prefrontal activity during walking in PD patients^12,19^, highlighting the role of the prefrontal cortex and higher cognitive functioning in gait in PD. Overground walking is a complex process that involves executive and cognitive functions^20^. A recent study revealed an association between the elevated prefrontal activity and poorer cognitive performance indicating a negative manifestation^8^. In the present work, the patients group showed lower levels of prefrontal cortex connectivity and significantly reduced prefrontal cortex connectivity during NW compared with DT at follow-up, that was closer to the observed pattern in controls. Clearly, even if TT reduces prefrontal activity to a certain degree, the cognitive processing during a more complicated task such as DT will require greater cognitive resources^19^. One can speculate that reduced prefrontal activity during NW might leave more capacity for cognitive processing on the more complex concurrent task, in a sense, improving efficiency. Our findings from the connectivity analysis, which represents the neural coupling in bilateral prefrontal activity during walking^21^, provide another dimensionality to this area compared to other hemoglobin-based fNIRS studies.

Recent studies suggested that the cerebellum plays a compensatory role in PD, and is associated with both motor and cognitive performance^22,23^. Based on our findings, enhancement of motor and cognitive pathways (*cerebellum* ⇀ *subcortical region* and *motor cortex* ⇀ *subcortical region* connectivity) were observed following TT (Fig. 3). The reduced ECs to the motor cortex (*subcortical region* ⇀ *motor cortex*; *brainstem* ⇀ *motor cortex*), and increased EC from the motor cortex (*motor cortex* ⇀ *subcortical region*) may suggest of less modulatory influence needed from other brain regions, and more pronounced information flow from the motor cortex to the subcortical region.

Despite not being statistically significant after FDR correction, a negative trend was detected between MDS-UPDRS-III scores and *prefrontal cortex* ⇀ *motor cortex* connectivity (Fig. 5a). This suggests that higher information flow from the prefrontal cortex to motor cortex is associated with better motor performance in PD patients. It is well established that the prefrontal cortex plays a critical role in executive functions, including the management of cognitive functions such as planning, working memory, and cognitive flexibility^24^. Various strategies have been found to be effective in compensating for gait impairments in patients with PD, including the use of external cues and motor imagery^25^. Even though effective compensatory strategies for motor impairment may vary between patients, our results (Fig. 5a) may suggest that there is a possible compensatory modulation of intrinsic brain connectivity between large-scale brain regions that target the motor cortex via the prefrontal cortex^26^.

In this study, a positive trend between *brainstem* ⇀ *subcortical region* connectivity and UPDRS-Total score (Fig. 5b) was also observed, suggesting that higher connectivity is related to poorer UPDRS-Total performance. According to a recent study, neuromodulatory brainstem systems may alter cortical population activity through a variety of mechanistic pathways directly or indirectly^27^. Dopamine is one of the major neuromodulators that travel from the brainstem to the subcortical regions. In patients with PD, dopamine loss in the substantia nigra of the midbrain may result not only in abnormalities in the neuromodulatory system, but also in dysfunction of the basal ganglia-brainstem pathway, which in turn can alter cortical activity in a broad range of ways^27^. Thus, it is not surprising that ascending directional connectivity from brainstem to subcortical region was found to associate with UPDRS-Total score which reflect cognitive, motor and autonomic functions. It should be noted, however, since patients with PD exhibited a lower level of connectivity in *brainstem* ⇀ *subcortical region* than healthy controls (Supplementary Table S2), the positive association with UPDRS-Total scores may imply a different mechanism for neuromodulation in the basal ganglia-brainstem pathway among PD patients. In addition, PD patients at baseline also exhibited significantly higher connectivity in *brainstem* ⇀ *motor cortex* compared to the control group. This elevated resting-state EC from the brainstem may suggest extensive modulatory effects originating from the brainstem, and it is likely responsible for the impairment during motor performance.

In terms of SVM models, several EC features are informative in both baseline and follow-up, implying a regional alteration following TT. Regarding the neural modulations underlying prefrontal activity during walking, we found specific EC patterns for the patient group that explained a substantial portion (PD patient at baseline: *R*^2^ = 0.63 ± 0.1; at follow-up: *R*^2^ = 0.71 ± 0.03) of the prefrontal cortex connectivity during NW. At baseline, subcortical and cerebellar regions played a major role in the network and the connected regions are responsible for both motor and cognitive functioning. As EC from cerebellum to the prefrontal cortex was the most informative feature in the network, it is also possible that the cerebellum, which engages variability of movement and motor learning, prompts the prefrontal cortex in the regulation of movements and the cognitive planning or programming during walking^28^. At follow-up, our data-driven approach showed the optimal SVR model trained on EC features that were similar to the baseline pattern, yet with two additional features and higher accuracy. Indeed, the additional features increase the dimensionality of the feature space but are more informative in terms of prediction. This suggests more regions or networks are engaged in motor performance, particularly prefrontal cortex related ECs that are move involved following TT.

Specifically, the EC pattern (Fig. 7) can distinguish the patients and controls with 91.05% mean accuracy. Within this pattern, each EC feature contains predictive information contributing to the separation of the two cohorts. The major EC features are related to the cerebral cortex and subcortical region, which are in line with the cortico-basal ganglia pathway. In addition, EC features that are informative to differentiate two groups often imply the difference between the two. Therefore, the two reciprocal connections *brainstem* ⇄ *motor cortex* and *cerebellum* ⇄ *prefrontal cortex* may also indicate the bidirectional impairment of the pathway between these brain regions in PD.

The motor pathway from *motor cortex* ⇀ *brainstem* often travels via the corticospinal pathway. Recent studies demonstrated the impaired corticospinal tract^29^ and alteration based on imaging measures^30^ in PD, indicating the structural changes of the corticospinal pathway. According to the Shapley value of *motor cortex* ⇀ *brainstem* (Fig. 7), this connectivity plays a more informative role in distinguishing the patients from healthy controls, reflecting patterns of altered connectivity in PD patients. Similarly, *cerebellum* ⇄ *prefrontal cortex* connections were recognized as the predictive features as well, implying the reciprocal connection may be pathologically altered in PD. Even though the cerebellum has been primarily regarded as a critical region for motor function^31^, its engagement in cognitive functions has been reinforced in the past years^32^. Since the fronto-cerebellar association in Parkinson’s disease still remains unclear, our results provide a new perspective regarding its potential role in compensatory processes following motor training in PD.

One limitation of the study is the relatively small number of subjects. Nevertheless, the study design and the application of different hemodynamic measurements (fNIRS and fMRI) from all the subjects provide a comprehensive assessment of functional changes following TT. It should also be noted that EC estimation and machine learning regression methods are complex measurement that may not map onto each other in a linear manner. The robustness and generalizability of the results can be enhanced by using both large-scale brain regions and machine learning model evaluations based on nested cross-validation. In terms of the interpretation, lower Shapley values cannot provide information on the redundancy of the feature. Since it considers only a distributed contribution to the model prediction by a given model, the least contribution might also be crucial to the prediction.

In conclusion, the present study demonstrated patterns of neural alterations within distinct brain networks in PD patients following TT. When the patients walk on the treadmill, where the treadmill belt can operate as an external cue to pace the gait, the task-dependent workload of cognitive processing in the prefrontal cortex is reduced. Ultimately, our findings suggest that TT may lead to recruitment of compensatory effects recruited from other brain regions to the motor cortex. Regarding the resting-state connectivity pattern underlying the walking performance, the cerebellum and its intrinsic connectivity with the brainstem and subcortical region, which demonstrated a strong association with the prefrontal activity during walking, constructed the fundamental outline of the pattern. Furthermore, the prefrontal-directed connectivity (from brainstem and subcortical region) may reflect brain reorganization in the alleviated prefrontal activity following the treadmill training.

## Methods

### Study participants

Nineteen patients clinically diagnosed with idiopathic PD (74.0 ± 6.6 years, 13 males, 6 females; disease duration 10.5 ± 6.8 years) from V­TIME project^33^, and 19 age- and sex-matched healthy controls (69.9 ± 5.9 years, 9 males, 10 females) were included in this study (Table 1). The general inclusion criteria for PD patients were (a) being able to walk at least 5 min unassisted, (b) taking stable medications for the past month and (c) taking antiparkinsonian medication. Participants were excluded if they had psychiatric comorbidity (e.g., major depressive disorder as determined by DSM-V criteria), clinical diagnosis of dementia or other clinically significant cognitive impairment (Mini­ Mental State Examination (MMSE) score < 24), a history of clinical stroke, clinically significant traumatic brain injury or other neurological disorder that could affect their performance (other than PD), any orthopaedic problems that may affect their gait or had an unstable medical condition, including cardiovascular instability. Recruitment was performed via reaching out to outpatient and related clinics of the geriatric medicine and neurology departments of the Tel Aviv Medical Center (TLVMC) in Tel Aviv, Israel. The study was approved by the institutional review board at TLVMC, and the study was performed in accordance with the Declaration of Helsinki guidelines. All participants gave written informed consent prior to participation.

### Clinical and cognitive assessments

Clinical and cognitive assessments were conducted at the start of the study (baseline). These assessments were performed with “ON” medication, approximately 1 hour after taking the medication. In this study, the clinical motor impairment and disability of PD was assessed using the UPDRS-III and the UPDRS-Total scores. The cognitive assessment included the Montreal Cognitive Assessment (MoCA)^34^ and Trails Making Test (TMT)^35^ to evaluate motor and executive function.

### Gait and balance assessments

Gait was measured at the start of the study (baseline) and after completion of the training (follow-up) using an electronic walkway with pressure sensors embedded in a Zeno walkway carpet (ProtoKinetics LLC, Havertown, Pennsylvania). Participants were asked to walk under two conditions each for 1 min along a 30-m walkway: normal walking (NW) and dual-task walking (DT), while calculating subtractions from a three-digit number sequentially. Stride length, stride mean time, and cadence were evaluated for each condition^36^.

### Training protocol

All the PD patients in the current study underwent a 6-week treadmill training program, as part of the active control group in the V-TIME project^33^. The training protocol is consistent with the previous literature and pilot studies^13,33^. All participants were trained 3 times a week. Each training session lasted about 45 minutes, and started with 5 minutes of “warm up” (only walking on the treadmill). The participants walked on the treadmill with a safety harness that prevented falls but did not provide body weight support. The healthy control group underwent the same assessment protocol but did not receive any training.

### fNIRS acquisition

Dynamic fluctuations in oxygenated hemoglobin (HbO) and hemoglobin (HHb) concentrations in prefrontal cortex were assessed using PortaLite fNIRS systems (Artinis Medical Systems, Elst, the Netherlands) with 3 infrared light transmitters and 1 receiver in a single probe at 2 wavelengths (763 and 855 nm). This system is a preferred choice for task-based fNIRS studies, as the wireless technology of PortaLite allows participants to move without the restriction of wires. Two probes, shielded from ambient light by black fabric covering, were placed on the right and left forehead of the participants. To avoid measuring the midline sinus, probes were positioned at 7% of the head circumference with 15% height of the nasion-inion distance from nasion to the left and right from the midline. The positions approximately target left and right Brodmann area 10, dorsolateral and anterior prefrontal cortex. The sampling rate was 10 Hz. Oxysoft software application version 3.0.52 (Artinis Medical Systems, Elst, the Netherlands) was used for data storage. We assessed prefrontal activity before and after the 6-week treadmill training program during NW and DT tasks. Every task was performed 5 times; each trial began with 20 s of silence, during which participants were instructed not to speak or move their heads. Following these 20 s, the instruction “start” or “start with [number]” was given (for subtraction in DT). After walking back and forth along a 30-m walkway for 30 s, the participants were instructed to stop and stand quietly for another 20 s. Participants were given rest periods between trials according to their needs. A participant was required to stand for at least one minute before each trial began in order to minimize blood pressure fluctuations following standing up. All tasks were conducted with “ON” medications approximately one hour after taking them.

### MRI acquisition

Whole-brain imaging data from all subjects were collected on a 3 T Signa Excite MRI scanner equipped with an 8-channel phased array head coil. Imaging data from the patients were measured both before and after 6-week TT program. The control group underwent MRI scanning at baseline only. T1-weighted images were acquired using a 3D spoiled gradient echo sequence with following parameters: repetition time (TR) = 59 ms; echo time (TE) = 3.6 ms; inversion time (TI) = 500 ms; flip angle (FA) = 8°; field of view (FoV) = 256 × 256 mm; number of slices = 162, voxel size = 0.98 × 0.98 × 1 mm^3^. Rs-fMRI data consisted of 266 volumes using single-shot echo-planar imaging (EPI) sequence with TR = 1700 ms; TE = 35 ms ; FA = 90°; FoV = 64 × 64 mm ; 30 axial slices with no gap ; voxel size = 3.1 × 3.1 × 3.5 mm^3^.

### fNIRS preprocessing

The fNIRS preprocessing steps were implemented in line with one of our previous study^37^, we first extracted raw data (light intensity) from the Artinis software (Oxysoft v3.0.52), and then converted it to delta optical density (OD)^38^. Next, moving standard deviation and spline interpolation methods^39^ were applied on the time series followed by wavelet artefact correction with parameters recommended by Molavi and Dumont^40^ to remove motion artefacts. Finally, the relative HbO and HHb concentration changes were calculated using Beer-Lambert Law^41^ on the cleaned OD data with a wavelength and age-dependent constant differential pathlength factor^42^. All fNIRS preprocessing were implemented in MATLAB (R2019a; MathWorks, Natick, Massachusetts, United States of America) using Homer2 toolbox^38^. Importantly, we did not use any filtering in the fNIRS preprocessing pipeline prior to connectivity analysis in order to avoid unwanted effects such as spurious connections^43^.

### MRI preprocessing

rs-fMRI data preprocessing steps were carried out following recommended guidelines^44,45^ in Statistical Parametric Mapping (SPM12) software. This included the following: (1) removal of the first five volumes to account for T1 ­relaxation effects, (2) head motion correction using rigid body translation and rotation, (3) slice timing correction, (4) co–registration to anatomical T1 image, (5) normalization to Montreal neurological institute (MNI) space using the deformation matrices obtained during MRI preprocessing using the CAT12 toolbox (Structural Brain Mapping Group, Jena University Hospital, Jena, Germany)^46^, (6) smoothing with a 6 mm full­width half maximum kernel, and (7) nuisance covariate regression (including six motion correction parameters, and averaged WM and CSF signals).

### rs-fMRI region of interests

To gain a conceptual and robust overview of EC changes across the brain regions, we avoided using small, compartmentalized brain areas. Instead, we selected large-scale regions of interest (ROIs) in light of maximal population level reproducibility and biological validity, while also accommodating for individual anatomic variability. Based on the alterations in large ­scale brain network associated with motor and non­motor symptoms of PD^47,48^, we selected five ROIs: the prefrontal cortex, motor cortex, cerebellum, brainstem and the subcortical region including the caudate nucleus, putamen, globus pallidus internus and externus, substantia nigra, thalamus and subthalamic nucleus. The detail of the atlas coordinates and sub­areas are listed in the supplementary material (Supplementary Table S1). To obtain a comprehensive brain atlas that covers all the ROIs, we combined four common atlases in this study. The prefrontal cortex and brainstem were extracted from the Harvard Oxford cortical atlas^49^, cerebellum was masked by the probabilistic cerebellar atlas with nonlinear normalization in FSL software^50^, the motor cortex was extracted from the Jülich atlas^51^ and subcortical region was defined according to the PD25 atlas^52^. All of the atlases were transformed to the MNI space before masking the brain region of the subjects. Finally, the voxels of each brain region were averaged as per rs-fMRI volume and extracted as the time series for further connectivity analysis.

### Time-resolved partial directed coherence

To analyze the directed connectivity at a specific frequency, we used time-resolved partial directed coherence (TPDC). Due to its insensitivity to indirect influences, it has been used for both fNIRS and fMRI studies^37,53^. The TPDC^54^ adopts the dual-extended Kalman filter^55^ to estimate time-dependent autoregressive coefficients. Subsequently, we used the Fourier transformation of the estimated time­-dependent multivariate autoregressive (MVAR) coefficients to calculate partial directed coherence (PDC)^56^. PDC from time­series *x*_*j*_ to *x*_*i*_ at each time point can be calculated by:

1 $$\pi _{i \leftarrow j}\left( f \right) = \frac{{|A_{ij}(f)|}}{{\sqrt {\mathop {\sum }\nolimits_{k = 1}^N |A_{kj}(f)|^2} }}$$

The PDC shows the strength of the connection in the frequency (*f*) domain based on the principle of Granger causality, where *A*_*ij*_ indicates the Fourier transformed MVAR coefficients and *N* refers to the number of the pairwise connection. After squaring the PDC value, the normalized value falls between 0 and 1. In the rs-fMRI time series, we extracted the frequency band of interest from 0.009 to 0.08 Hz and averaged across each time point to obtain robust connectivity values between brain regions. For consistency of the estimation in the present study, we applied TPDC on the fNIRS dataset for both left and right probes. Since no left-right difference was present in the activation (*P* > 0.23) for all groups, we averaged bidirectional EC in prefrontal cortex for further analysis. Each trial of the walking task was computed separately and then averaged across each trial. The extracted frequency range for fNIRS was 0.009 to 0.08 Hz^37,57^.

### Support vector machine methods

In machine learning, SVM methods are supervised learning algorithms that use kernel methods to transform the given data samples to high dimensional space. Based on the transformations, it constructs a hyperplane to fit the dataset and corresponding outputs, which is widely used in classification and regression problems. We choose SVM methods due to its effusiveness where the number of dimensions is greater than the number of observations, and recent history of impressive neuroimaging results^58,59^. In this work, we applied SVM models on the EC features derived from rs-fMRI to predict the prefrontal connectivity computed from the fNIRS time series during walking.

Several types of kernels have been commonly used in SVM models to map the features to the high-dimensional space, such as the polynomial kernel and the Gaussian kernel. Given the nonlinearity of brain activity^60^, we used the Gaussian kernel which is also known as radial basis function (RBF):

2 $$k_\sigma \left( {x,x^\prime } \right) = exp\left( - \frac{{\parallel x - x^\prime \parallel ^2}}{{2\sigma ^2}}\right)$$

In terms of SVM optimization^61^, the proper choice of the kernel coefficient and regularization parameters is crucial to the SVM performance. Nevertheless, tuning these values to find the optimal hyper­parameter is out of the scope of the current study. In this work, the SVM models were carried out using the Statistics and Machine Learning Toolbox in MATLAB (R2019a; MathWorks, Natick, Massachusetts, United States of America) with default kernel coefficients and regularization parameters. The complete description of default fitting parameters can be found in MATLAB documentation^62^.

### Feature selection and model validation

To reduce the model complexity and remove redundant predictors^63^, feature selection methods such as sequential feature selector and recursive feature elimination are able to reduce the number of features effectively and are computationally inexpensive. However, these methods may not be robust because the feature combinations were not thoroughly evaluated. Therefore, we used exhaustive feature selector (EFS)^63^. In EFS, each feature subset is trained with SVM, meaning it loops over every possible combination of features.

In this study, SVM models were trained on resting-state ECs (20 ECs between five ROIs) in patients at baseline and follow-up, which gives us a total number of feature combinations as 2^20^ = 1.048576*10^6^. In order to avoid the bias in performance evaluation and leakage of test data, each model was validated with nested cross–validation, in which a second *k*–fold cross-validation loop is used within the training data^64^. This approach is preferred for model evaluation especially for small sample size^65^. In addition, the configuration of *k* in the cross-validation algorithm is a bias–variance trade­off. When *k* gets larger, the re­sampling validation/test set gets smaller causing higher variance. When *k* gets smaller, the bias of the technique becomes lower. Since there is no formal rule that defines the proper *k* to be used in cross–validation, we used a common choice of *k* = 5 for both outer and inner loop of the nested cross-validation in this work^63,66^. For the model evaluation, we calculate R-squared for regression models and correct classification accuracy, sensitivity, and specificity in classification models. The optimal models were selected based on the best average performance with minimal standard errors across each fold of cross-validation.

### Feature contribution

In order to determine the impact and the contribution of each feature on the predicted output of SVM, we used the Shapley value based explanation known as Kernel SHapley Additive exPlanations (SHAP)^67^. Recently, the Shapley value based explanation has been used in several studies to explain the prediction of machine learning models on neuroimaging data^66,68^.

In the context of prediction modelling, the Shapley value explains the contribution of the features to the predicted value of the model. Each feature of the observation can be seen as a “player” in a cooperative game where the prediction is the “pay-out”. This procedure will identify contributions of the EC features that are associated with the predicted prefrontal activity during walking.

The explanation of individual observation by SHAP greatly increases the transparency and enables us to reveal the impact of the EC features. In the present work, the global explanation is represented by the mean absolute Shapley value for each feature across individual observations. Features with large absolute Shapley value are viewed as important features^66,69,70^. Given that the interpretation based on one single model may be biased, we averaged the mean absolute Shapley value over each fold in the cross-validation for less variance and biased results. The Shapley value implementation for SVM models was carried out using the Statistics and Machine Learning Toolbox in MATLAB (R2021a; MathWorks, Natick, Massachusetts, United States of America).

### Statistical analysis

If not stated otherwise, all analysis were performed using custom written R scripts (version 4.1.2 Bird Hippie). Figures were produced using ggplot2 package^71^. Paired-samples *t*-test was performed to probe the changes in behavioral measures pre-and post TT. Non-parametric two-way ANOVA^72^ was applied on average prefrontal connectivity during walking with between-subject factor: group/training (2 levels: 1. patients at baseline vs controls, and 2. patients at baseline vs follow-up) and within-subject factor: condition (2 levels: normal walking and dual-task walking). The level of statistical significance against the null-hypothesis was set to *p* < 0.05 (two-tailed, false discovery rate (FDR) correction). One-sample Wilcoxon signed-rank test was used to assess the resting-state EC changes at baseline and follow-up in the patient group; the Mann–Whitney U test was performed to examine the differences of resting-state ECs between the controls and patients.

### Correlation analysis

In order to examine the relationship between the resting-state ECs in the patient group at baseline and follow-up, correlation analysis was performed on those resting-state ECs that were statistically significant from the aforementioned statistical tests. Normality was assessed by Shapiro–Wilk test. As resting-state ECs were not normally distributed dataset, we calculated Spearman’s correlation coefficients on the EC difference between baseline and follow-up in the patients using the equation:

3 $${{\Delta }}\,EC^i = EC_{baseline}^i-EC_{follow - up}^i$$

To further explore the association between intrinsic brain connectivity and PD-specific disease severity, we calculated correlation coefficients between UPDRS scores and resting-state ECs in the patient group at baseline. Since UPDRS scores are normally distributed datasets, non-normal distributed ECs were transformed using power transformation^73^ and Pearson correlation analysis was applied. In terms of multiple comparison, the correction method FDR was applied.

## Supplementary information

Supplemental material

## Data Availability

The dataset for the present study is not publicly available as it contains information that could breach research participant privacy/consent, but is available from the corresponding authors upon reasonable requests from qualified researchers, within the limitations of the provided informed consent. Every request will be reviewed by Tel Aviv University, Tel Aviv, Israel, and the requesting researcher will need to sign a data access agreement after approval.

## Code availability

Image analysis was carried out using SPM 12 (https://www.fil.ion.ucl.ac.uk/spm/). The custom code that supports the connectivity analysis of this study are publicly available at Github (https://github.com/kenhding/fNIRS-fMRI-connectivity).
