# Supplementary material for: Treadmill training in Parkinson’s disease is underpinned by the interregional connectivity in cortical-subcortical network
Source: NPJ Parkinsons Dis. 2022 Nov 11;8:153. doi: 10.1038/s41531-022-00427-3 (PMC9652466; doi:10.1038/s41531-022-00427-3)
Supplement: Supplementary file 1 — Supplemental material [file 41531_2022_427_MOESM1_ESM.pdf]

Supplementary Material

Supplementary Table S1. region of interest included in the atlas and their affiliation to the investigated brain areas (merged region) in the current study.

**Brain region and atlas**	**sub brain areas**
Cerebellum<br>(cerebellar atlas<br>with nonlinear<br>normalization in FSL[1])	Left Crus I<br>Left Crus II<br>Left I-IV<br>Left IX<br>Left V<br>Left VI<br>Left VIIb<br>Left VIIIa<br>Left VIIIb<br>Left X<br>Right Crus I<br>Right Crus II<br>Right I-IV<br>Right IX<br>Right V<br>Right VI<br>Right VIIb<br>Right VIIIa<br>Right VIIIb<br>Right X<br>Vermis Crus I<br>Vermis Crus II<br>Vermis IX<br>Vermis VI<br>Vermis VIIb<br>Vermis VIIIa<br>Vermis VIIIb<br>Vermis X
Prefrontal cortex<br>(Harvard-Oxford atlas[2])	Frontal Medial Cortex Left<br>Frontal Medial Cortex Right<br>Frontal Orbital Cortex Left<br>Frontal Orbital Cortex Right<br>Frontal Pole Left<br>Frontal Pole Right<br>Inferior Frontal Gyrus, pars opercularis Left<br>Inferior Frontal Gyrus, pars opercularis Right<br>Inferior Frontal Gyrus, pars triangularis Left<br>Inferior Frontal Gyrus, pars triangularis Right

	Middle Frontal Gyrus Left<br>Middle Frontal Gyrus Right<br>Superior Frontal Gyrus Left<br>Superior Frontal Gyrus Right
Motor cortex<br>(Jülich atlas[3])	Juxtapositional Lobule Cortex (formerly Supplementary Motor Cortex) L<br>Juxtapositional Lobule Cortex (formerly Supplementary Motor Cortex) R<br>Postcentral Gyrus L<br>Postcentral Gyrus R<br>Premotor cortex BA6 L<br>Premotor cortex BA6 R<br>Primary motor cortex BA4a L<br>Primary motor cortex BA4a R<br>Primary motor cortex BA4p L<br>Primary motor cortex BA4p R<br>Primary somatosensory cortex BA1 L<br>Primary somatosensory cortex BA1 R<br>Primary somatosensory cortex BA2 L<br>Primary somatosensory cortex BA2 R<br>Primary somatosensory cortex BA3a L<br>Primary somatosensory cortex BA3a R<br>Primary somatosensory cortex BA3b L<br>Primary somatosensory cortex BA3b R<br>Secondary somatosensory cortex Parietal operculum OP1 L<br>Secondary somatosensory cortex Parietal operculum OP1 R<br>Secondary somatosensory cortex Parietal operculum OP2 L<br>Secondary somatosensory cortex Parietal operculum OP2 R<br>Secondary somatosensory cortex Parietal operculum OP3 L<br>Secondary somatosensory cortex Parietal operculum OP3 R<br>Secondary somatosensory cortex Parietal operculum OP4 L<br>Secondary somatosensory cortex Parietal operculum OP4 R
Subcortical structures<br>(PD25 atlas[4])	Left caudate<br>Left globus pallidus externa<br>Left globus pallidus interna<br>Left putamen<br>Left substantia nigra<br>Left subthalamic nucleus<br>Left thalamus<br>Right caudate<br>Right globus pallidus externa<br>Right globus pallidus interna<br>Right putamen<br>Right Substantia nigra

	Right subthalamic nucleus<br>Right thalamus
Brainstem<br>(Harvard-Oxford atlas[2])	Brainstem

Supplementary Table S2. Resting-state effective connectivity summary and group comparison with Wilcoxon signed-rank test/Mann-Whitney-Test.

	Mean(Median)			P-value		
Connectivity	Baseline (patients)	Follow-up (patients)	Controls	Baseline v.s Follow-up	Baseline v.s Controls	Follow-up v.s Controls
Cerebellum → Brainstem	0.359(0.264)	0.339(0.366)	0.331(0.304)	0.407	1	0.62
Prefrontal cortex → Brainstem	0.383(0.335)	0.327(0.292)	0.354(0.37)	0.084	0.839	0.342
Motor cortex → Brainstem	0.367(0.332)	0.331(0.288)	0.357(0.346)	0.434	0.369	0.374
Subcortical region → Brainstem	0.348(0.371)	0.289(0.266)	0.348(0.337)	0.123	0.434	0.221
Brainstem → Cerebellum	0.065(0.048)	0.058(0.042)	0.054(0.045)	0.722	0.772	0.729
Prefrontal cortex → Cerebellum	0.105(0.102)	0.097(0.09)	0.101(0.072)	0.522	0.506	0.557
Motor cortex → Cerebellum	0.098(0.097)	0.104(0.082)	0.095(0.073)	0.758	0.487	0.599
Subcortical region → Cerebellum	0.082(0.072)	0.107(0.083)	0.088(0.081)	0.162	0.603	0.94
Brainstem → Prefrontal cortex	0.029(0.026)	0.026(0.024)	0.034(0.024)	0.554	0.729	0.407
Cerebellum → Prefrontal cortex	0.057(0.046)	0.058(0.052)	0.068(0.044)	0.492	0.795	0.845
Motor cortex → Prefrontal cortex	0.051(0.043)	0.08(0.056)	0.084(0.076)	0.135	0.065	0.845
Subcortical region → Prefrontal cortex	0.051(0.052)	0.06(0.058)	0.056(0.041)	0.308	0.544	0.537
Brainstem → Motor cortex	0.049(0.043)	0.031(0.027)	0.05(0.029)	**0.044**	0.296	0.358
Cerebellum → Motor cortex	0.067(0.045)	0.079(0.078)	0.075(0.066)	0.434	0.839	0.599
Prefrontal cortex → Motor cortex	0.093(0.08)	0.076(0.078)	0.082(0.073)	0.407	0.469	0.729
Subcortical region → Motor cortex	0.085(0.089)	0.06(0.058)	0.076(0.066)	**0.044**	0.09	0.407
Brainstem → Subcortical region	0.036(0.027)	0.053(0.034)	0.049(0.044)	0.943	0.154	0.133
Cerebellum → Subcortical region	0.053(0.046)	0.072(0.045)	0.079(0.067)	0.522	**0.023**	0.257
Prefrontal cortex → Subcortical region	0.071(0.065)	0.084(0.068)	0.083(0.079)	0.286	0.283	1
Motor cortex → Subcortical region	0.061(0.056)	0.072(0.06)	0.086(0.069)	0.619	**0.049**	0.342

Supplementary Table S3. Correlation analysis between UPDRS and resting-state effective connectivity

Connectivity	UPDRS-III		UPDRS-Total	
	r	*p*	*r*	*p*
Brainstem → Cerebellum	0.330735	0.166646	0.311919	0.193586
Brainstem → Prefrontal cortex	-0.06882	0.779518	0.087366	0.722106
Brainstem → Motor cortex	-0.08027	0.743912	0.179651	0.461768
Brainstem → Subcortical region	0.40906	0.082029	0.577496	**0.009618**
Cerebellum → Brainstem	0.014114	0.954268	-0.06107	0.803851
Cerebellum → Prefrontal cortex	0.169495	0.48787	0.173017	0.478739
Cerebellum → Motor cortex	-0.14683	0.548618	0.067092	0.784929
Cerebellum → Subcortical region	0.239566	0.323234	0.429807	0.066263
Prefrontal cortex → Brainstem	-0.12579	0.607853	-0.19167	0.431815
Prefrontal cortex → Cerebellum	0.180637	0.459274	0.269571	0.264388
Prefrontal cortex → Motor cortex	-0.45718	**0.049074**	-0.12915	0.598241
Prefrontal cortex → Subcortical region	0.26011	0.282165	0.149887	0.540232
Motor cortex → Brainstem	0.020028	0.935139	-0.05711	0.816371
Motor cortex → Cerebellum	0.193135	0.428241	0.194936	0.423862
Motor cortex → Prefrontal cortex	0.299633	0.212661	0.342145	0.151621
Motor cortex → Subcortical region	0.279781	0.246009	0.100878	0.681135
Subcortical region → Brainstem	0.110094	0.653665	-0.08675	0.723987
Subcortical region → Cerebellum	-0.09996	0.683893	0.035655	0.884781
Subcortical region → Prefrontal cortex	-0.24674	0.308509	-0.06947	0.777497
Subcortical region → Motor cortex	-0.08124	0.740922	-0.15048	0.538598

References

[1] J. Diedrichsen, S. Maderwald, M. Küper, M. Thürling, K. Rabe, E. Gizewski, M. E. Ladd, and D. Timmann, "Imaging the deep cerebellar nuclei: a probabilistic atlas and normalization procedure," *Neuroimage*, vol. 54, no. 3, pp. 1786–1794, 2011.

[2] N. Makris, J. M. Goldstein, D. Kennedy, S. M. Hodge, V. S. Caviness, S. V. Faraone, M. T. Tsuang, and L. J. Seidman, "Decreased volume of left and total anterior insular lobule in schizophrenia," *Schizophrenia research*, vol. 83, no. 2-3, pp. 155–171, 2006.

[3] S. B. Eickhoff, K. E. Stephan, H. Mohlberg, C. Grefkes, G. R. Fink, K. Amunts, and K. Zilles, "A new spm toolbox for combining probabilistic cytoarchitectonic maps and functional imaging data," *Neuroimage*, vol. 25, no. 4, pp. 1325–1335, 2005.

[4] Y. Xiao, V. Fonov, S. Bériault, F. Al Subaie, M. M. Chakravarty, A. F. Sadikot, G. B. Pike, and D. L. Collins, "Multi-contrast unbiased mri atlas of a parkinson's disease population," *International journal of computer assisted radiology and surgery*, vol. 10, no. 3, pp. 329–341, 2015.
